# An Unusual Case of Type 1 Narcolepsy in an Ethiopian Patient

**DOI:** 10.4314/ejhs.v31i1.23

**Published:** 2021-01

**Authors:** Asefa Mekonnen, Gregory Stimac

**Affiliations:** 1 Rockville Internal Medicine Group Department of Sleep Medicine, 1201 Seven Locks Rd Suite 111, Rockville, ND; 2 Georgetown University School of Medicine, 3800 Reservoir Rd NW, 20007

**Keywords:** Narcolepsy, polysomnogram, Ethiopia, sleep disorders

## Abstract

**Background:**

Narcolepsy is a chronic disabling central neurological disorder of daytime hypersomnia. It is categorized into two subtypes-type 1 (N1) and type 2 (N2). Symptoms of N1 commonly include excessive daytime sleepiness (EDS), cataplexy, sleep paralysis, hypnogogic/hypnopompic hallucinations, and disturbed nighttime sleep. Ethnic differences have been observed, but they have not been reported in an Ethiopian patient to date.

**Case Detail:**

We report a 39-year-old Ethiopian patient with type 1 narcolepsy whose diagnosis was delayed for three decades despite severe symptoms. Her quality of life was significantly impaired and included EDS, sleep fragmentation, and depression. The mean sleep latency (MSL) for five naps was 1.3 minutes. Sleep-onset rapid eye movement (REM) periods (SOREMPs) were present in all five nap periods. HLA-typing and a CSF hypocretin level testing were not performed. Modafinil 300mg was prescribed, which improved her quality of life.

**Conclusion:**

In developing countries where diagnostic studies are not available, practitioners should pay special attention to a detailed history and look for classic symptoms of narcolepsy to establish an early diagnosis and improve quality of life.

## Introduction

Narcolepsy is a lifelong central neuroglial disease of daytime sleepiness. It is believed to have a worldwide prevalence, although ethnic differences in its presentation are observed (1). Recent advances categorize the disease as type I (N1) or type II (N2) narcolepsy. N1 symptoms include excessive daytime sleepiness (EDS), cataplexy, sleep paralysis, hypnogogic/hypnopompic hallucinations, and disturbed nighttime sleep. Its causation requires both a genetic predisposition and an event-triggered destruction of the lateral hypothalamic hypocretin-producing neurons resulting in cerebrospinal fluid (CSF) hypocretin deficiency, possibly an autoimmune process incited by an environmental factor, infection, or exposure to vaccines (1,2). N1 initially presents at a younger age, frequently before the second decade, with insidious progression of EDS. In spite of its disabling symptoms and psychosocial impact, it often takes 10–15 years for patients to receive the correct diagnosis (3).

## Case Report

A 39-year-old Ethiopian-American woman presented with a history of severe EDS since childhood. She recalled falling asleep in class, during conversations, and walking with her mother holding hands. She reported fragmented, unrefreshing sleep and occasional hallucinations on awakenings. Although a diagnosis of narcolepsy was not made in Ethiopia, where she spent her childhood, dextroamphetamine was prescribed. She relocated to the United States but interrupted her college education due to EDS, sleep fragmentation, and depression. She occasionally experienced loss of muscle control triggered by laughter, primarily in her hands, and experienced episodes of automatic behavior, mostly while driving. Daytime naps were refreshing. Her past medical history was significant for attention deficit disorder treated with dextroamphetamine 10mg, which partly improved her EDS. She denied head trauma, snoring, witnessed apneas, restless legs symptoms, or any other complaints. Family and social histories were unexceptional.

The patient appeared healthy with normal vitals and physical exam. She weighed 87.5kg, measured 157.5cm, and BMI was 35.3kg/m^2^. Laboratory analysis including urine toxicology and TSH were normal. She scored 24/24 on the Epworth Sleepiness Scale (ESS). An overnight PSG followed by Multiple Sleep Latency Test (MSLT) was conducted an American Academy of Sleep Medicine (AASM) accredited facility in Rockville, Maryland, United States. This demonstrated normal architecture without pathology (Table 1). The mean sleep latency (MSL) for five naps was 1.3 minutes with sleeponset rapid eye movement (REM) periods (SOREMPs) in all five nap periods (Table 2). HLA-typing and a CSF hypocretin level were not performed due to her classic presentation and reported cataplectic symptoms. She was diagnosed with N1. Dextroamphetamine was discontinued, and modafinil 200mg was prescribed (4). She noted significant improvements in morning sleepiness but had residual afternoon sleepiness, which resolved with an additional modafinil 100mg. On followup, she reported significantly improved quality of life and daytime alertness.

**Table 1:** Diagnostic PSG

Characteristics	Value
Total Recording time (minutes)	407.0
Total sleep time (minutes)	323.5
Sleep efficiency	79.5%
Sleep onset Latency (minutes)	7.0
Wake after sleep onset (minutes)	75.5
REM Latency (minutes)	45.5
Apnea/hypopnea index (events/hour)	0
Mean SpO2	98%
Sleep stage distribution	
NREM 1	10.4%
NREM 2	56.1%
NREM 3	18.4%
REM	15.1%
Periodic Limb Movement Index (events/hour)	1.9

NREM: non-rapid eye movement; PSG: Polysomnography/Polysomnogram; REM: rapid eye movement

**Table 2:** MSLT done following nocturnal PSG

Characteristics	Nap 1	Nap 2	Nap 3	Nap 4	Nap 5
Sleep Onset Latency (minutes)	0.5	1.5	2.0	1.0	1.5
REM Onset Latency (minutes)	1.5	2.5	8.5	4.5	5.0

MSLT: Multiple Sleep Latency Test, PSG: Polysomnography/Polysomnogram; REM: rapid eye movement

## Discussion

The diagnosis of narcolepsy requires comprehensive histories and examinations focusing on severity of daytime sleepiness, sleep attacks, nocturnal sleep quality, sleep-onset, awakening hallucinations, sleep paralysis, cataplexy, automatic behaviors, and a detailed family history followed by diagnostic testing. Our case demonstrates a significant delay in diagnosis and appropriate therapy for narcolepsy. Her triggered loss of muscle tone is consistent with cataplexy and a diagnosis of N1. Our patient posed a serious risk to herself and the public while driving. However, her cataplectic symptoms were infrequent, so we deferred treatment. Nocturnal PSG followed by daytime MSLT demonstrating pathological sleepiness, as indicated by a MSL <8 minutes with ≥2 SOREMPs, suggests narcolepsy.

Patients with N1 show markedly decreased levels of hypocretin in their CSF, and 98% share HLA-DQB1*06:02, a gene implicated in many different autoimmune diseases (1). HLA-DQB1* 06:02, seen in almost all patients with N1, has a prevalence of 39.4% among African Americans, 23.8% of Caucasians, 16.1% of Latinos, and 8.8% of Asians (2). African Americans are less likely to have the pathognomonic symptom of cataplexy, are more likely to present earlier, and with a higher ESS score (2). No data exist concerning the prevalence of HLA-DQB1*06:02 or degree of N1 cataplexy in Ethiopians.

Management of narcolepsy includes pharmacological, behavioral, and psychosocial interventions. Pharmacological intervention is used to treat symptoms of EDS, nighttime sleep fragmentation, and cataplexy. Modafinil, armodafinil, pitolissant, solriamfetol sodiun oxybate (gamahydroxybutrate) and novel wake-promoting agents have improved the care of narcolepsy in the last decade (4,5). Serotonin reuptake inhibitors, tricyclic antidepressants, and L-carnitine (6), are also being studied as pharmacological additions (4,5).

This patient was prescribed dextroamphetamine initially, which improved her EDS. A higher dose could have helped the patient, but given the long term side-effects and the luck of selectivity with dextroamphetamine (7), most narcolepsy patients in areas where options for therapy exist have been switched to modafinil, armodafinil, sodium oxybate, and other novel wake-promoting agents in recent years (8). We do understand that in resource-limited countries for testing and therapy choices, the use of higher dose of dextroamphetamine could have helped her symptoms.

In developing countries where diagnostic studies are not available, practitioners should conduct detailed histories and identify classic symptoms of narcolepsy to establish a diagnosis. Future management of narcolepsy may include immunotherapy, hypocretin-based therapy, endocrine therapy, and stem cell transplantation. Patient and family education, sleep hygiene, strategic napping, cognitive behavioral therapy, depression treatment, and investigation into future therapy can improve quality of life.
